# Impact of Alien Chromosome Introgression from *Thinopyrum ponticum* on Wheat Grain Traits

**DOI:** 10.3390/plants14193072

**Published:** 2025-10-04

**Authors:** Shuwei Zhang, Yu Zhang, Ting Hu, Linying Li, Zihao Wang, Linyi Qiao, Lifang Chang, Xin Li, Zhijian Chang, Peng Zhang, Xiaojun Zhang

**Affiliations:** 1 College of Agriculture, Shanxi Agricultural University, Taiyuan 030031, China; zshuwei@sxau.edu.cn (S.Z.); 20232196@stu.sxau.edu.cn (Y.Z.); 20233132@stu.sxau.edu.cn (T.H.); 20233147@stu.sxau.edu.cn (L.L.); 20233085@stu.sxau.edu.cn (Z.W.); qiaoly1988@126.com (L.Q.); changlifang@sxau.edu.cn (L.C.); lixin@sxau.edu.cn (X.L.); wrczj@126.com (Z.C.);; 2 Key Laboratory of Sustainable Dryland Agriculture (Co-Construction by Ministry and Province), Ministry of Agriculture and Rural Affairs, Taiyuan 030031, China

**Keywords:** chromosome variation, fluorescence in situ hybridization, alien chromosome, chromosome transmission rate, grain traits, wheat

## Abstract

Structural variation (SV) serves as a fundamental driver of phenotypic diversity and environmental adaptation in plants and animals, significantly influencing key agronomic traits in crops. Common wheat (*Triticum aestivum* L.), an allohexaploid species, harbors extensive chromosomal SVs and distant hybridization-induced recombination events that provide critical resources for genetic improvement. This study utilizes non-denaturing fluorescence in situ hybridization (ND-FISH) and oligonucleotide multiplex probe-based FISH (ONPM-FISH) to analyze the karyotypes of 153 BC_1_F_4_–BC_1_F_6_ lines derived from the hybrid line Xiaoyan 7430 and common wheat Yannong 1212. The results revealed that Xiaoyan 7430 carries 8 alien chromosome pairs and 20 wheat chromosome pairs (lacking 6B), and Yannong 1212 contains 21 pairs of wheat chromosomes. The parental lines exhibited presence/absence variations (PAVs) on chromosomes 2A, 6A, 5B, 1D, and 2D. Chromosomal variations, including numerical chromosomal variation (NCV), structural chromosomal variation (SCV), and complex chromosomal variation (CCV), were detected in the progeny lines through ONPM-FISH analysis. The tracking of alien chromosomes over three consecutive generations revealed a significant decrease in transmission frequency, declining from 61.82% in BC_1_F_4_ to 26.83% in BC_1_F_6_. Telosomes were also lost during transmission, declining from 21.82% in BC_1_F_4_ to 9.76% in BC_1_F_6_. Alien chromosome 1J^S^, 4J, and 6J exhibited the highest transmission stability and were detected across all three generations. Association analysis showed that YN-PAV.2A significantly affected the length/width ratio (LWR) and grain diameter (GD); YN-PAV.6A, XY-PAV.6A, and PAV.5B increased six grain traits (+2.25%~15.36%); YN-PAV.1D negatively affected grain length (GL) and grain circumference (GC); and XY-PAV.2D exerted positive effects on thousand-grain weight (TGW). Alien chromosomes differentially modulated grain characteristics: 1J^S^ and 6J both reduced grain length and grain circumference; 1J^S^ increased LWR; and 4J negatively impacted TGW, grain width (GW), GD, and grain area (GA). Meanwhile, increasing alien chromosome numbers correlated with progressively stronger negative effects on grain traits. These findings elucidate the genetic mechanisms underlying wheat chromosomal variations induced by distant hybridization and their impact on wheat grain traits, and provide critical intermediate materials for genome design breeding and marker-assisted selection in wheat improvement.

## 1. Introduction

Genomic variation is widespread in plants and animals and can induce phenotypic or functional changes [1,2,3]. The occurrence of genomic variation encompasses multiple forms, including single-nucleotide polymorphisms (SNPs), insertion/deletion variations (Indels), translocations, inversions, presence/absence variations (PAVs), copy number variations (CNVs), and numerical chromosomal variations (NCVs) [4,5,6]. Recent studies have demonstrated associations between genomic variation and key agronomic traits [7,8]. For example, structural variations influence grain size and disease resistance in rice (*Oryza sativa*) [9,10] and modulate flowering time and stay-green traits in rapeseed (*Brassica napus*) [11,12]. Furthermore, structural variations play critical roles in crop adaptation and resistance to biotic/abiotic stresses [13,14,15], such as enhancing freezing tolerance in barley (*Hordeum vulgare*) [16] and conferring glyphosate tolerance in maize (*Zea mays*) [17]. Thus, the in-depth exploration of structure variation–trait associations is essential for deciphering the genetic basis of phenotypic diversity.

Common wheat is an allohexaploid species whose origin involved two polyploidization events across three species (*Aegilops* and *Triticum*), accompanied by frequent chromosomal variations during long-term evolution [18,19,20,21]. Extensive studies indicate that common wheat underwent intense artificial selection during domestication, with chromosomal variations conferring adaptation to regional environments and superior agronomic traits being retained [22,23]. For instance, Cavanagh et al. [24] analyzed SNPs in 2994 hexaploid wheat accessions worldwide, identifying selective variations associated with flowering time and climatic adaptation, and demonstrated the targeted selection of alleles for specific traits across geographical regions. Similarly, Wu et al. [25] conducted chromosomal structural analyses of randomly selected landraces and elite breeding lines, including 123 accessions from southern China and 110 cultivars from northern China. They revealed that the T6VS·6AL translocation predominated in southern China, while T1RS·1BL was the most prevalent structural variation in northern China. Thus, characterizing chromosomal structural variations and their phenotypic/functional impacts is essential for understanding wheat domestication and accelerating cultivar development.

Fluorescence in situ hybridization (FISH), which employs fluorescently labeled probes for chromosomal karyotyping, is widely applied in wheat research for chromosome identification, structural variation analysis, alien chromosome detection, germplasm development, and gene localization [26,27]. For instance, ND-FISH has been employed to characterize rust-resistant wheat–*Thinopyrum intermedium* translocation lines [28] and *Fusarium* head blight-resistant wheat– *Psathyrostachys huashanica* addition lines [29]. Furthermore, oligonucleotide probe multiplex (ONPM)-based FISH enables precise wheat karyotyping and efficiently identifies chromosomal polymorphisms and structural variations [23,25,30]. For example, Zhao et al. [31] conducted precise karyotyping of 262 Chinese wheat mini-core collections and 281 Shanxi wheat cultivars using ONPM-FISH, detecting 139 structural variations at 230 loci including translocations, pericentric inversions, presence/absence variations (PAVs), and copy number variations (CNVs). Subsequent genome-wide association analysis between phenotypic traits and these structural variations revealed that Mr2D-7 was significantly associated with thousand-grain weight (TGW), grain length (GL), grain width (GW), and grain thickness (GT); Mg2B-6 significantly increased TGW, GW, and GT, whereas Mg1D-2 negatively affected all three. Similarly, Zhang et al. [32] identified ten PAVs, one CNV, and one 1RS·1BL translocation in wheat cultivars Jinmai 47 and Jinmai 84. Association analysis with agronomic traits demonstrated that PAV.2D increased grain number per spike (GNS), while PAV.4A.1, PAV.6A, and PAV.6B influenced GL, GT, spike length (SL), and TGW. These findings collectively demonstrate that chromosomal structural variations exert substantial genetic effects on the agronomic performance of wheat.

Distant hybridization between wheat and related genera induces frequent chromosomal structural variations. Previous studies indicate that alien chromosome introgression can induce extensive genetic variations in wheat, including numerical and structural chromosomal changes [33,34,35]. For example, Hu et al. [36] employed Giemsa-C banding, genomic in situ hybridization (GISH), and fluorescence in situ hybridization (FISH) to analyze the karyotype of wheat–*Thinopyrum ponticum* partial amphiploid 7430, revealing a variable chromosome number ranging from 53 to 56. Liu et al. [37] analyzed translocation lines derived from crosses between *Psathyrostachys huashanica* and common wheat, revealing not only a double translocation (T3DS·3DL-4NsL and T3DL-4NsS) involving 4Ns alien chromosome and wheat 3D chromosome but also translocations in wheat chromosomes 2A and 4A. Similarly, Wang et al. [38] identified structural variations in both wheat and alien chromosomes across 59 wheat–rye derived lines. Results indicate that distant hybridization between wheat and related genera induces frequent chromosomal structural variations during the process. Jiang et al. [39] analyzed the karyotypes of 2384 M1-M3 plants from the cross between the wheat–*Thinopyrum intermedium* addition line Th93-1-6 and the wheat cultivar MY11, and finally identified 37 types of chromosomal variations. Introducing elite genes from related genera into wheat through distant hybridization serves as an effective strategy for wheat breeding improvement and novel germplasm development. For instance, the development of alien chromosome introgression lines involving genera such as *Thinopyrum* [40], *Secale* (rye) [41], *Aegilops* [42], and *Agropyron* [43] has been extensively reported. However, due to the typically large size of introgressed alien chromosomal segments, beneficial genes are often accompanied by undesirable ones, leading to genetic drag. This results in poor agronomic performance of the developed materials, limiting their direct application in wheat breeding, and there is still limited research in this area now. Therefore, investigating the genetic effects and transmission characteristics of alien chromosome introgressions on wheat agronomic traits is crucial for harnessing elite genes from related genera while mitigating negative impacts, thereby advancing breeding in wheat chromosome engineering.

In this study, we analyzed the karyotypes of Xiaoyan 7430 (a wheat–*Thinopyrum ponticum* partial amphidiploid) and Yannong 1212 (common wheat) via ND-FISH and ONPM-FISH with multiple probes, and characterized chromosomal variations in BC_1_F_4_–BC_1_F_6_ progeny derived from their hybridization and backcrossing. The main objectives are as follows: (1) to identify variations in wheat chromosomal structure induced by alien introgression; (2) to elucidate the transmission patterns of alien chromosomes in wheat backgrounds; and (3) to reveal the genetic effects of alien chromosomes on wheat grain traits. This study will provide new genetic resources for improving wheat through distant hybridization breeding.

## 2. Results

### 2.1. Chromosomal Composition of Xiaoyan 7430 and Yannong 1212

**Figure 1:**
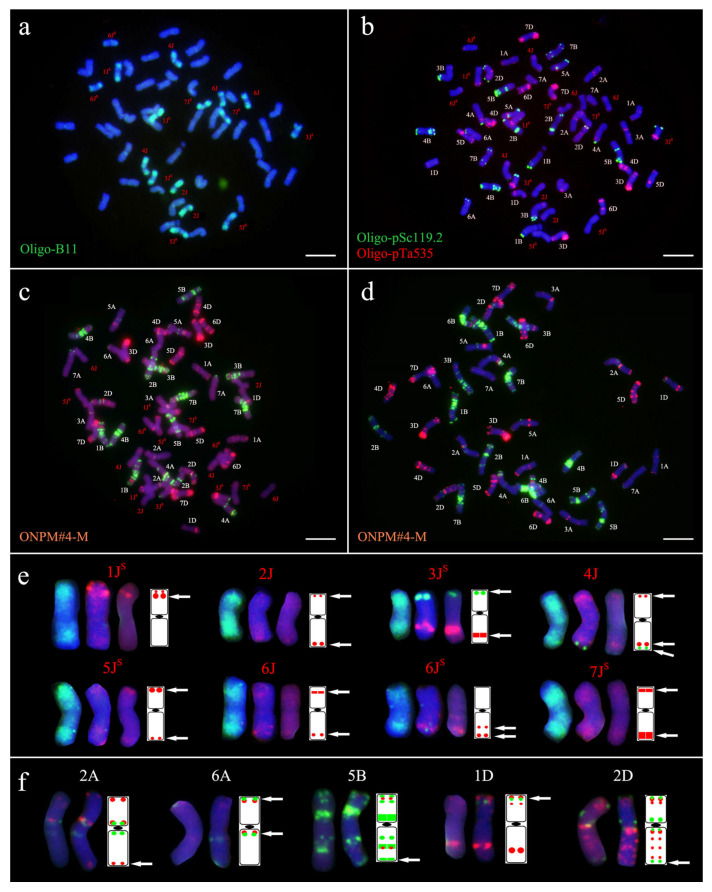
Karyotyping of the mitotic metaphase and diagram of Xiaoyan 7430 and Yannong 1212. (**a**,**b**): Sequential ND-FISH of Xiaoyan 7430 using the following probes: (**a**) Oligo-B11 (green); (**b**) Oligo-pSc119.2 (green) and Oligo-pTa535 (red). (**c**,**d**): ONPM-FISH of Xiaoyan 7430 (**c**) and Yannong 1212 using the ONPM # 4-M probe (red: Oligo-pTa535, Oligo-pAs1-1 and Oligo-pTa71; green: Oligo-pSc119.2 and Oligo-(GAA)_10_). Bars: 10 μm. (**e**): Karyotype and diagram of 8 alien chromosomes of Xiaoyan 7430, with each probe group in the order of Oligo-B11 (green), Oligo-pSc119.2 (green)+Oligo-pTa535 (red), ONPM # 4-M (red: Oligo-pTa535, Oligo-pAs1-1 and Oligo-pTa71; green: Oligo-pSc119.2 and Oligo-(GAA)_10_), and the respective diagrams. The white arrows indicate the location of signals on the alien chromosomes. (**f**): Structural variations on 2A, 6A, 5B, 1D, and 2D of Xiaoyan 7430 (**left**) and Yannong 1212 (**middle**) using the ONPM # 4-M probe (red: Oligo-pTa535, Oligo-pAs1-1 and Oligo-pTa71; green: Oligo-pSc119.2 and Oligo-(GAA)_10_), and the respective diagrams (**right**). The white arrows indicate the location of the differential signals.

The karyotyping of Xiaoyan 7430 using Oligo-B11, Oligo-pSc119.2, and Oligo-pTa535 probes revealed a chromosome composition of 40 wheat chromosomes and 16 alien chromosomes, lacking one pair of chromosome 6B. With reference to the karyotype of Xiaoyan 7430 established by Jiang et al. [44] using Oligo-B11, Oligo-pSc119.2, and Oligo-pTa535, the eight pairs of alien chromosomes were identified as 1J^S^, 2J, 3J^S^, 4J, 5J^S^, 6J, 6J^S^, and 7J^S^. (Figure 1a,b). Following the wheat karyotyping framework established with ONPM#4 by Du et al. [30], reference karyotypes for Xiaoyan 7430 and Yannong 1212 were constructed using ONPM#4-M. As shown in Figure 1c, the probes produced distinct signal patterns across all 56 chromosomes of Xiaoyan 7430, enabling clear differentiation of all 40 wheat chromosomes and 16 alien chromosomes. The alien chromosomes exhibited distinct signal patterns (Figure 1e). 1J^S^ showed an intense red signal subterminally on its short arm; both 2J and 6J^S^ displayed strong red signals at the terminus of their long arms, though 6J^S^ also exhibited a prominent red signal in the mid-region of its long arm, while 2J featured a weak red signal at the short-arm terminus; 3J^S^ and 6J both carried strong red signals proximal to the long-arm terminus, yet 3J^S^ presented a distinct green signal at the short-arm terminus, while 6J had strong red signals subterminally on the short arm; 4J demonstrated intense red and green signals at the long-arm terminus, accompanied by a weak red signal at the short-arm terminus; and 5J^S^ and 7J^S^ each possessed strong red signals at the short-arm terminus, but 5J^S^ displayed a weak red signal at the long-arm terminus, whereas 7J^S^ exhibited a strong red signal subterminally on the long arm. For Yannong 1212, the ONPM#4-M probe resolved all 42 wheat chromosomes with distinct patterns (Figure 1d). Comparative analysis identified five structural variations between parental lines (Figure 1f). Yannong 1212 exhibited YN-PAV.2A (distal red signal on 2AL), YN-PAV.6A (centromere-proximal green signal on 6AL), YN-PAV.5B (terminal green signal on 5BL), and YN-PAV.1D (terminal green signal on 1DS), which were all absent in Xiaoyan 7430. Conversely, Xiaoyan 7430 possessed XY-PAV.6A (terminal green signal on 6AS) and XY-PAV.2D (terminal green signal on 2DL), which were not detected in Yannong 1212.

### 2.2. Analysis of Chromosomal Variations in BC_1_F_4_–BC_1_F_6_-Derived Lines

#### 2.2.1. Karyotype Characterization of Derived Lines

ONPM-FISH analysis of 153 BC_1_F_4_–BC_1_F_6_-derived lines from crosses between Xiaoyan 7430 and Yannong 1212 revealed rich signal polymorphisms across all chromosomes. Comparative analysis with parental karyotypes enabled precise identification of wheat and alien chromosomes in progeny (Figure S1–S3). These lines exhibited an extensive range of chromosomal variations, categorized into three primary classes: (1) numerical chromosomal variations (NCVs), encompassing wheat chromosome additions/deletions and alien chromosome introgressions; (2) structural chromosomal variations (SCVs), including signal presence/absence variations, chromosomal segment deletions, translocations, isochromosomes, and telosomes; and (3) complex chromosomal variations (CCVs), combining both numerical and structural alterations (Tables S1–S3).

#### 2.2.2. Analysis of Chromosomal Variations in Derived Progenies

**Figure 2:**
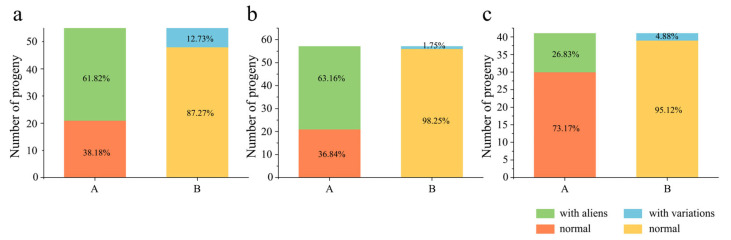
Chromosomal variation ratios of BC_1_F_4_–BC_1_F_6_-derived lines: (**a**): BC_1_F_4_; (**b**): BC_1_F_5_; and (**c**): BC_1_F_6_. (A) Proportion of alien chromosomes and (B) proportion of chromosomal variations in the population.

Karyotyping of 55 BC_1_F_4_ lines revealed numerical chromosomal variations (NCVs) ranging from 42 to 48 chromosomes, with 34 lines (61.82%) carrying alien chromosomes and 7 (12.73%) exhibiting wheat chromosome additions/deletions (Figure 2a). In 57 BC_1_F_5_ lines, NCV ranged from 42 to 46 chromosomes: 36 lines (63.16%) harbored alien chromosomes (comparable to BC_1_F_4_), while only 1 (1.75%) showed wheat chromosome deletions—a marked reduction from BC_1_F_4_. For BC_1_F_6_ (41 lines), NCV spanned 41 to 46 chromosomes, with merely 11 lines (26.83%) retaining alien chromosomes (significantly reduced versus earlier generations) and 2 (4.88%) displaying wheat chromosome additions/deletions, indicating the progressive loss of alien chromosomes in advanced generations.

**Figure 3:**
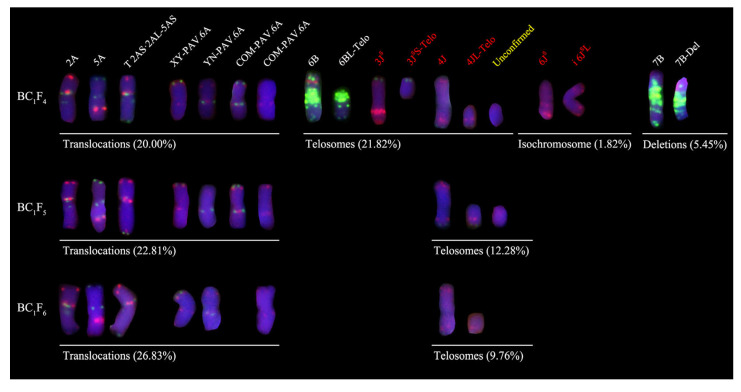
Types of chromosomal variations in BC_1_F_4_–BC_1_F_6_-derived lines. Red signals show Oligo-pTa535, Oligo-pAs1-1 and Oligo-pTa71; green indicates Oligo-pSc119.2 and Oligo-(GAA)_10_.

Chi-square tests on PAV recombination across five differential chromosomes revealed segregation distortion for four chromosomes (excluding 5B) in BC_1_F_4_ (Table 1). Translocations were observed in 11 lines (20.00%), including T2AS·2AL-5AS (fusion of 2A long arm and 5A short arm terminal signals) and two novel 6A recombinants: COM-PAV.6A (retaining both parental green signals) and NULL-PAV.6A (losing both green signals) (Figure 3). Additionally, 12 lines (21.82%) harbored telosomes, 1 (1.82%) exhibited an isochromosome translocation, 3 (5.45%) showed segmental deletions, and 19 (34.55%) displayed complex chromosomal variations (CCV) (Table S1). In BC_1_F_5_, segregation distortion persisted for four chromosomes, excluding 5B (Table 1). No segmental deletions or isochromosomes were detected, but 13 lines (22.81%) carried translocations, 7 (12.28%) retained telosomes (indicating progressive loss), and 12 (21.05%) exhibited CCV—showing significantly reduced complexity versus BC_1_F_4_ (Figure 3, Table S2). By BC_1_F_6_, segregation normalized for chromosomes 2A, 5B, and 2D but remained distorted for 6A and 1D (Table 1). No segmental deletions or isochromosomes occurred, while 11 lines (26.83%) showed translocations (increased proportion), 4 (9.76%) retained telosomes (further loss), and only 5 (12.20%) displayed CCV, demonstrating progressively reduced variation complexity with advancing generations (Figure 3, Table S3).

### 2.3. Alien Chromosome Transmission in Derived Progenies

**Figure 4:**
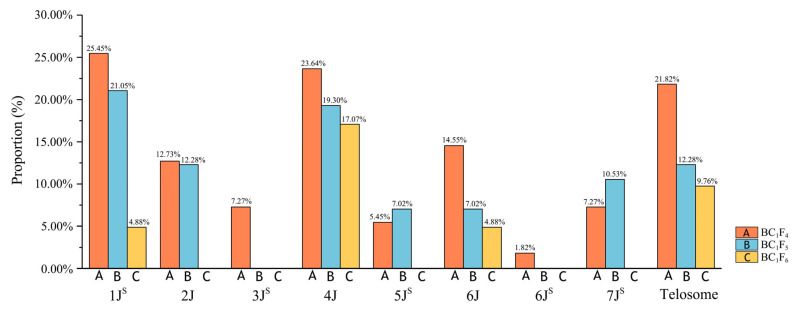
Alien chromosome transmission rate in BC_1_F_4_–BC_1_F_6_-derived lines. Transmission rates in A: BC_1_F_4_; B: BC_1_F_5_; C: BC_1_F_6_.

The cytogenetic characterization of BC_1_F_4_ lines (Table S1) revealed that all eight alien chromosome pairs were distributed across different lines as monosomic, disomic, and telosomic types. Notably, one monosomic telosome and six disomic telosomes exhibited indistinguishable signal patterns, precluding precise chromosomal assignment. The telosomes were analyzed collectively. Transmission frequencies for individual alien chromosomes were quantified as follows: 1J^S^ (25.45%) > 4J (23.64%) > 6J (14.55%) > 2J (12.73%) > 7J^S^ (7.27%) = 3J^S^ (7.27%) > 5J^S^ (5.45%) > 6J^S^ (1.82%). These results indicate enriched retention of 1J^S^ and 4J in progeny, while 6J^S^ showed minimal transmission. Telosomes occurred at a high frequency of 21.82% (Figure 4). No 3J^S^ or 6J^S^ chromosomes/fragments were detected in BC_1_F_5_ progenies (Table S2). The six remaining alien chromosomes (1J^S^, 2J, 4J, 5J^S^, 6J, and 7J^S^) introgressed as monosomic, disomic, or telosomic types. Transmission frequencies ranked 1J^S^ (21.05%) > 4J (19.30%) > 2J (12.28%) > 7J^S^ (10.53%) > 6J (7.02%) = 5J^S^ (7.02%), mirroring BC_1_F_4_ trends and confirming the superior stability of 1J^S^/4J. Telosomes persisted at 12.28% (Figure 4). Only 1J^S^, 4J, and 6J were detected (as disomic or telosomic) in BC_1_F_6_ (Table S3), with transmission frequencies of 4J (17.07%) > 1J^S^ (4.88%) = 6J (4.88%). The other five alien chromosomes (2J, 3J^S^, 5J^S^, 6J^S^, and7J^S^) were undetectable. Telosome transmission declined to 9.76% (Figure 4), demonstrating progressive attrition of alien chromatin across advanced generations.

### 2.4. Analysis of Genetic Effects of Chromosomal Variations on Grain Traits

#### 2.4.1. Effects of Chromosomal Variations on Grain Traits

Grain traits including TGW, GL, GW, LWR, GD, GC, GC, GA, and grain roundness (GR) were evaluated for all derived lines across BC_1_F_4_–BC_1_F_6_ generations over three consecutive years. Association analysis between chromosomal variations and grain traits was performed, with variations showing consistently significant effects (*p* < 0.05) on the same trait across all three years, and was thus deemed to exert biologically meaningful effects. Three-year averages were used to calculate effect values for these variations (Table 2 and Tables S4–S11). Key results are detailed below.

YN-PAV.2A significantly affected LWR and GD, with effect values of −4.63% and +2.77%, respectively. Both YN-PAV.6A and XY-PAV.6A influenced seven grain traits (except GR): YN-PAV.6A increased GL by 1.81%, while XY-PAV.6A increased it by 3.01%; conversely, COM-PAV.6A reduced GL (−0.91%) (Table 2). XY-PAV.6A increased GW (+1.22%), whereas YN-PAV.6A and COM-PAV.6A decreased it (−4.63% and −1.18%, respectively). YN-PAV.5B exerted positive effects on six traits (excluding LWR and GR), with effect values ranging from +2.25% to +15.36% (Table 2), indicating its potential for enhancing grain yield in breeding programs. Structural variations on chromosome 1D negatively affected GL (−0.22 mm) and GC (−0.59 mm). XY-PAV.2D increased TGW (+1.58 g) but reduced GL (−2.82%) and GA (−0.19%). Alien chromosomes also modulated grain traits (Table 2): 1J^S^ increased LWR (+3.62%) but decreased GL (−0.09%) and GC (−1.12%); 4J significantly affected TGW, GW, LWR, GD, and GA (while it positively influenced LWR (+11.88%), it negatively impacted the other four traits (−15.47%, −8.71%, −3.96%, and −7.59%, respectively)); and 6J reduced GL (−0.29 mm) and GC (−0.50 mm). Collectively, alien chromosomes predominantly exerted negative effects on grain characteristics.

#### 2.4.2. Analysis of Dose Effects of Alien Chromosomes on Grain Traits

**Figure 5:**
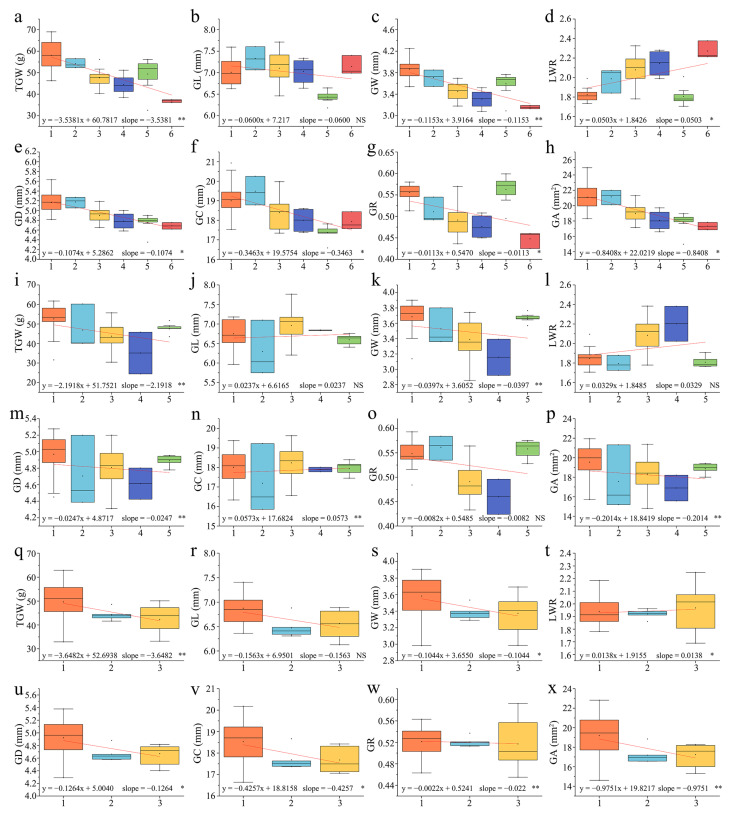
Association analysis between the number of alien chromosomes and different grain traits in BC_1_F_4_–BC_1_F_6_-derived lines. Association analysis of 8 grain traits in (**a**–**h**): BC_1_F_4_; (**i**–**p**): BC_1_F_5_; and (**q**–**x**): BC_1_F_6_. The horizontal axis represents the number of alien chromosomes, and the vertical axis represents different grain traits. * indicates significant differences at the *p* < 0.05 level; ** indicates significant differences at the *p* < 0.01 level. NS: not significant.

To assess the potential effects of alien chromosome dosage on grain traits, derivative lines were grouped by ascending number of alien chromosomes and their impact on various agronomic characteristics was evaluated. In BC_1_F_4_, seven traits (excluding LWR) exhibited progressively stronger negative effects with increasing alien chromosome numbers (Figure 5a–h). BC_1_F_5_ displayed the same patterns, in which GL, LWR, and GC showed increasing trends with low trendline coefficients, while TGW, GW, GD, GR, and GA decreased markedly (Figure 5i–p). In BC_1_F_6_, seven traits (excluding LWR) demonstrated negative effects correlating with higher alien chromosome counts (Figure 5q–x). These results indicate that increased alien chromosome dosage correlates with progressively stronger negative impacts on wheat grain traits. Thus, mitigating the detrimental effects of alien chromosomes on agronomic traits should be prioritized when developing distant hybridization breeding materials.

#### 2.4.3. Impact of Special Wheat Chromosomal Variations on Grain Traits

**Figure 6:**
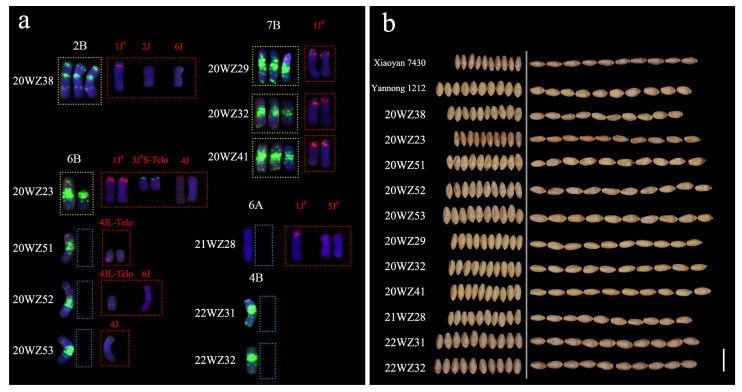
Special wheat chromosomal variations in 20WZ38, 20WZ23, 20WZ51, 20WZ52, 20WZ53, 20WZ29, 20WZ32, 20WZ41, 21WZ28, 22WZ31, and 22WZ32 and their grain morphology. (**a**): Special wheat chromosomal variations in different lines, red signals show Oligo-pTa535, Oligo-pAs1-1 and Oligo-pTa71; green indicates Oligo-pSc119.2 and Oligo-(GAA)_10_, the yellow dashed box indicates variations in the wheat chromosome, the blue dashed box indicates deletion of the wheat chromosome, and the red dashed box indicates an alien chromosome. (**b**): Grain morphology. Bar: 1 cm.

Karyotypic analysis of derivative lines identified 11 lines with wheat chromosomal variations including monosomic, trisomic, and telosomic. They were compared with parental lines to assess the effects of chromosomal variations on grain traits, with complete karyotypes presented in Figure S5. Line 20WZ38 (46 chromosomes) exhibited a trisomic variation in chromosome 2B along with single copies of 1J^S^, 2J, and 6J (Figure 6 and Figure S5a). Compared to the wheat parent, seven traits (excluding LWR) decreased by 4.06–28.30% (Table S12). Four BC_1_F_4_ lines displayed abnormal chromosome 6B configurations (Figure 6 and Figure S5b–e). 20WZ23 (48 chromosomes) contained one complete 6B, one 6BL telosome, a pair of 1J^S^, paired 3J^S^ short-arm telosomes, and a pair of 4J; 20WZ51, 20WZ52, and 20WZ53 all lacked one 6B chromosome but carried alien segments, and 20WZ51 had a pair of 4J long-arm telosomes; 20WZ52 obtained one 4J long-arm telosome and one 6J; and 20WZ53 obtained one 4J. These four lines showed a 12.54–41.11% reduction in TGW but a 6.94–9.81% increase in GL (Table S12). Lines 20WZ29, 20WZ32, and 20WZ41 (45 chromosomes each) showed trisomic variations of 7B with partial terminal deletions on one long arm resulting in a loss of terminal green signals, and carried paired 1J^S^ (Figure 6 and Figure S5f–h). Compared to the wheat parent, TGW and GW decreased by 19.95–22.62% and 15.62–16.19%, respectively, while GL increased by 6.94–11.44% (Table S12). Line 21WZ28 (44 chromosomes) lacked one 6A chromosome while carrying one 1J^S^ and paired 5J^S^ (Figure 6 and Figure S5i), showing reductions in seven grain traits except length-to-width ratio (Table S12). Lines 22WZ31 and 22WZ32 (both 41 chromosomes) each lacked one wheat chromosome 4B and were devoid of alien chromosomes (Figure 6 and Figure S5j,k), exhibiting significant TGW reduction (9.13–9.14%) but minimal changes in other grain traits (Table S12). This suggests that monosomic variation of 4B reduces TGW, while the absence of alien chromosomes preserves grain traits.

## 3. Discussion

### 3.1. Genetic Characteristics of Chromosome Variations in Wheat–Thinopyrum Ponticum-Derived Lines

The introgression of alien chromosomes from wild relatives into wheat through distant hybridization induces extensive chromosomal variations in wheat [34,35]. Investigating transmission patterns and genetic characteristics of alien chromosomes in wheat backgrounds is crucial for effectively transferring desirable genes from related species. Blanco et al. [45] observed severe loss of alien chromosomes in self-pollinated progeny of durum wheat–*Dasypyrum villosum* monosomic addition lines, with transmission rates of monosomic and disomic *Dasypyrum* chromosomes varying widely. This study revealed that *Thinopyrum ponticum* chromosomes 1J^S^, 4J, and 6J exhibited higher transmission stability in wheat backgrounds, persisting across three consecutive generations, while the other five alien chromosome pairs showed lower transmission rates and were lost during generational advancement. These results demonstrate that alien chromosome introgression simultaneously induces variations in both wheat and alien chromosomes, though the underlying mechanisms driving these variations require further investigation.

Chromosomal structural variations significantly influence meiotic recombination, with recombination hotspots in wheat showing associations with gene-rich regions [46]. Zhao et al. [47] identified novel karyotypes on chromosome 2A in doubled haploid (DH) progeny of two wheat cultivars using multiplex oligonucleotide probes, attributing these to meiotic recombination. Similarly, this study detected distinct FISH signal polymorphisms on chromosome 6A between parental lines. In derived progenies, two novel recombinant karyotypes were identified, one retaining both parental green signals (COM-PAV.6A) and another lacking both signals (NULL-PAV.6A), indicating that meiotic recombination generated these structural variants. This suggests that the reorganization of tandem repeat arrays may lead to structural variations in wheat [48]. During evolution, genetic interactions, cytoplasmic effects, genetic background divergence, and environmental factors collectively cause segregation distortion of parental genetic material [49,50,51,52]. The analysis of structural variation frequencies in derived lines revealed pronounced segregation distortion for chromosomes 2A, 6A, 1D, and 2D in BC_1_F_4_ and BC_1_F_5_ generations, with preferential transmission of Yannong 1212 alleles. In BC_1_F_6_, distortion persisted only for 6A and 1D—it was significantly reduced but still skewed toward Yannong 1212. We propose that this distortion primarily stems from genomic instability induced by introgressed alien chromosomes, while the bias toward the wheat parent suggests additional influences from cytoplasmic effects and gene interactions [53,54,55]. Nevertheless, studies on alien chromosome-induced segregation dynamics remain scarce, warranting deeper investigation into the mechanisms underlying structural variation transmission and distortion.

### 3.2. Genetic Effects of Chromosomal Variations on Grain Traits

Wheat grain traits including TGW, GL, GW, LWR, GD, GC, GR, and GA are critical yield components governed by complex genotype–environment interactions [56,57,58]. Previous studies localized grain-size-related genes to chromosomes 2A, 2B, 2D, 3A, 3D, and 6A [59,60,61,62,63], and identified QTLs for grain length, width, and weight on 1A, 2A, 2B, 2D, 3B, 4B, 4D, 5A, 5B, 6A, 7A, 7B, and 7D [63,64,65,66,67]. Our trait–variation association analysis revealed that structural variations on 2A significantly affected GD and LWR. Variations on 6A influenced nearly all grain traits. YN-PAV.5B exerted positive effects on TGW, GL, GW, LWR, GD, GC, and GA. Variations on 1D and 2D reduced GL, though XY-PAV.2D increased TGW. These results indicate that the structural variations analyzed are functionally linked to grain traits, suggesting polymorphic chromosomal regions may harbor grain-related genes. Cytological screening for advantageous structural variations could therefore enhance grain yield. Introgression of small alien segments has proven effective for wheat improvement [68,69,70]. For example, Sun et al. [71] developed translocation lines 7PT-A18 and 7PT-B4 carrying small segments of *Agropyron cristatum* chromosome 7P, significantly increasing GL, GW, and TGW. In this study, alien chromosomes 1J^S^, 4J, and 6J also influenced grain traits, but predominantly exerted detrimental effects: 1J^S^/6J reduced GL and GC, and 4J decreased TGW, GW, GD, and GA. Dosage analysis further demonstrated that increasing alien chromosome numbers intensified negative impacts on grain traits. The analysis of lines with special wheat chromosomal variations confirmed pronounced negative effects of alien chromosomes on grain traits. For instance, line 20WZ23, carrying four alien chromosomes and two alien telosomes, exhibited reductions in six grain traits compared with the wheat parent, particularly a 41.11% decrease in TGW. Lines 20WZ38 and 21WZ28, each harboring three alien chromosomes, both showed diminished values in seven grain traits. In contrast, lines 22WZ31 and 22WZ32, without any alien chromosomes, displayed minimal differences in grain traits compared to the wheat parent (Table S12). These results demonstrate that a higher alien chromosome dosage increases the likelihood of detrimental traits masking beneficial effects, ultimately impairing wheat grain performance. Therefore, to achieve effective exploitation of wild relatives in the genetic improvement of wheat, distant hybridization breeding must prioritize developing small-segment introgression lines to localize beneficial alien fragments, followed by cloning advantageous genes to unlock their breeding potential [72,73].

## 4. Materials and Methods

### 4.1. Materials

The plant materials used in this study included the partial amphidiploid Xiaoyan 7430, derived from *Thinopyrum ponticum* and provided by the Crop Science Institute, Shanxi Academy of Agricultural Sciences, Taiyuan, and the common wheat cultivar Yannong 1212, provided by the Yantai Academy of Agricultural Sciences, Yantai. A total of 153 BC_1_F_4_–BC_1_F_6_ derivative lines were developed through hybridization and backcrossing between these two parents. Xiaoyan 7430, created by crossing decaploid *Thinopyrum ponticum* with wheat followed by backcrossing, exhibits desirable agronomic traits including multi-disease resistance and large spikes with extended length. Yannong 1212 is a nationally registered cultivar adapted to the Yellow-Huai Valley Winter Wheat Zone of China that features compact plant architecture, plump kernels, and strong lodging resistance. The BC_1_F_4_–BC_1_F_6_ population was established through crossing Xiaoyan 7430 with Yannong 1212 to generate F_1_ hybrids, followed by backcrossing with Yannong 1212 and advancing generations via single-seed descent. All materials were maintained at the Shanxi Key Laboratory of Crop Genetics and Molecular Improvement.

### 4.2. Plant Cultivation

The plant materials, including Xiaoyan 7430, Yannong 1212, and the BC_1_F_4_–BC_1_F_6_-derived lines, were cultivated at the Dongyang Experimental Station solar greenhouse in Jinzhong City, Shanxi Province (37°33′ N, 112°40′ E, altitude 800 m), during three consecutive growing seasons: 2020–2021, 2021–2022, and 2022–2023. Each year, 40–60 lines were randomly selected from the derivative progeny for root-tip chromosome karyotyping. After the collection of root-tip samples, the plants were transplanted to the solar greenhouse and grown until harvest. The greenhouse was arranged with rows of 1 m length and 0.3 m spacing, with five plants transplanted per row. Due to failed germination of some seeds or seedling mortality after transplantation, a total of 55, 57, and 41 plants were ultimately obtained for this study in the three respective seasons.

### 4.3. Preparation of Root-Tip Mitotic Chromosomes

Root-tip mitotic chromosome preparations were produced following the method described by Lang et al. [74] with minor optimizations. The detailed procedure is as follows:

Seeds were placed embryo-down on moist filter paper in Petri dishes and incubated in a constant-temperature growth chamber for 2 days. When the roots reached 1.5–2 cm in length, root tips were excised, transferred to moistened 1.5 mL centrifuge tubes, and treated with nitrous oxide (N_2_O) at 0.8–1.2 MPa for 2 h. The treated roots were then fixed in pre-chilled 90% acetic acid for 10 min, rinsed three times with sterile water, and the meristematic regions were dissected. Root tips were enzymatically digested in a solution containing 1% pectinase and 2% cellulase at 37 °C for 1 h. After triple washing with 70% ethanol, the root tips were gently macerated and then vortexed vigorously. The homogenate was centrifuged at 4000 rpm for 3 min, resuspended in 28–42 μL of glacial acetic acid, and vortexed again. Suspensions were dropped onto pre-cleaned microscope slides, placed in humid chambers for air-drying, and examined under a microscope to identify cells with well-dispersed chromosomes.

### 4.4. Fluorescence In Situ Hybridization (FISH)

Non-denaturing fluorescence in situ hybridization (ND-FISH) was performed according to Fu et al. [75] with the following protocol:

Probes were thoroughly mixed with hybridization buffer, and aliquots of the mixture were pipetted onto pre-marked microscope slides. Coverslips were applied, and slides were incubated in a humidified chamber at 37 °C for 2 h. Post-hybridization, the slides were washed in 2× SSC for 5 min, air-dried, counterstained with DAPI (4′,6-diamidino-2-phenylindole), and covered with coverslips for 10 min. Chromosomes were visualized using an Olympus BX53F fluorescence microscope equipped (Tokyo, Japan) with a SPOT™ cooling Color CCD camera. All oligonucleotide probes were synthesized by Shanghai Generay Biotech Co., Ltd. (Shanghai, China) Oligo-B11: Designed from *Thinopyrum*-specific LTR sequence B11 [76], enabling specific hybridization to *Thinopyrum ponticum* and *Thinopyrum intermedium* chromosomes. Probe ONPM#4-M: Adapted from Du et al. [30], comprising TAMRA-labeled Oligo-pTa535, Oligo-pAs1-1, Oligo-pTa71, and FAM-labeled Oligo-pSc119.2 and Oligo-(GAA)_10_. This set accurately identifies all wheat chromosomes and generates discriminative signals on alien chromosomes (Table 3).

For sequential FISH assays requiring probe signal removal, the following stripping protocol was employed: Slides with previously hybridized probe signals were immersed in 1× PBS solution to facilitate coverslip detachment. Subsequently, the slides underwent three sequential 5 min washes in graded ethanol solutions: first in 70% ethanol, followed by 90% ethanol, and finally in 100% anhydrous ethanol. After thorough air-drying, the slides were ready for the next round of hybridization. This procedure effectively eliminated residual fluorescence signals while preserving chromosomal integrity for subsequent probe applications.

### 4.5. Grain Trait Evaluation

Grain traits were evaluated for BC_1_F_4_-, BC_1_F_5_-, and BC_1_F_6_-derived lines and their parental materials as follows:

Seeds from individual plants were manually threshed. Approximately 300 intact grains per line were randomly selected, and an automated grain analyzer (Xinliangtian Technology Co., Ltd., Shenzhen, China) was utilized to measure eight traits: thousand-grain weight (TGW), grain length (GL), grain width (GW), length/width ratio (LWR), grain diameter (GD), grain circumference (GC), grain roundness (GR), and grain area (GA). Each measurement was replicated three times, and mean values were calculated for subsequent analysis.

### 4.6. Data Analysis

Chromosomal images were processed and analyzed using Adobe Photoshop CS6. Structural variation and agronomic trait data were collated and preliminarily analyzed using Microsoft Excel 2016. Analysis of variance (ANOVA) and significance tests (*p* < 0.05) were performed using SPSS Statistics 17.0. Graphical representations of data were generated using OriginPro 9.1.

## 5. Conclusions

Integrated non-denaturing fluorescence in situ hybridization (ND-FISH) with alien- and wheat-specific probes enabled the detailed karyotyping of Xiaoyan 7430 and common wheat Yannong 1212. The results confirmed that Xiaoyan 7430 carried 40 wheat chromosomes and 16 alien chromosomes, lacking one pair of chromosome 6B, while Yannong 1212 possessed a complete complement of 42 wheat chromosomes. The subsequent precise karyotyping of parental lines and BC_1_F_4_–BC_1_F_6_-derived progeny using multiplex oligonucleotide probes (ONPM) identified structural variations on chromosomes 2A, 6A, 5B, 1D, and 2D, which exhibited significant segregation distortion across generations. The derived lines harbored extensive chromosomal variations—including numerical (NCV), structural (SCV), and complex (CCV) types—demonstrating that alien chromosomes induce substantial genomic restructuring in both wheat and alien chromatin. Transmission analysis revealed the superior stability of alien chromosomes 1J^S^, 4J, and 6J in the Yannong 1212 background. Association studies linking chromosomal variations to eight grain traits demonstrated the significant effects of polymorphic regions on five wheat chromosomes and three alien chromosomes. Notably, alien chromosomes predominantly exerted negative effects on grain characteristics, with increasing alien chromosome dosage intensifying these detrimental impacts. The results elucidate the genetic effects of alien chromosomes on eight wheat grain traits and their transmission patterns in wheat backgrounds, thereby providing critical intermediate materials for the use of grain-related elite genes from related genera in wheat breeding improvement.

## Figures and Tables

**Table 1:** Segregation ratios of structural variants in BC_1_F_4_–BC_1_F_6_-derived lines.

Population	Chr.	Yannong 1212 Types	Xiaoyan 7430 Types	Translocation Type 3	Translocation Type 4	χ ^2^	*p*
BC_1_F_4_	2A	19	33	3		14.511	0.000 ***
	6A	41	6	3	5	27.128	0.000 ***
	5B	27	28			0.036	0.850
	1D	45	10			11.77	0.000 ***
	2D	46	9			13.378	0.000 ***
BC_1_F_5_	2A	31	24	2		17.223	0.000 ***
	6A	40	6	5	6	22.027	0.000 ***
	5B	30	27			0.035	0.852
	1D	53	4			25.024	0.000 ***
	2D	48	9			14.445	0.000 ***
BC_1_F_6_	2A	13	20	8		2.286	0.319
	6A	30	3		8	14.126	0.000 ***
	5B	21	20			0.049	0.825
	1D	34	7			9.332	0.002 **
	2D	24	17			0.443	0.506

Note: ** indicates significant differences at the *p* < 0.01 level; *** indicates significant differences at the *p* < 0.001 level.

**Table 2:** Effect values of chromosomal variations on different grain traits.

Trait	Chr.	Structural Variation/Alien	Yannong 1212 Type	Xiaoyan 7430 Type	Effect Value
TGW	6A	YN-PAV.6A	48.06	53.75	−10.59%
	6A	XY-PAV.6A	52.16	53.75	−2.94%
	6A	COM-PAV.6A	52.09	53.75	−3.09%
	5B	YN-PAV.5B	53.15	46.08	15.36%
	2D	XY-PAV.2D	49.92	51.50	3.16%
	4J	4J	43.41	51.36	−15.47%
GL	6A	YN-PAV.6A	6.86	6.74	1.81%
	6A	XY-PAV.6A	6.94	6.74	3.01%
	6A	COM-PAV.6A	6.68	6.74	−0.91%
	5B	YN-PAV.5B	6.93	6.78	2.25%
	1D	YN-PAV.1D	6.83	7.05	−3.14%
	2D	XY-PAV.2D	6.91	6.71	−2.84%
	1J^S^	1J^S^	6.82	6.82	−0.09%
	6J	6J	6.59	6.88	−4.15%
GW	6A	YN-PAV.6A	3.51	3.68	−4.63%
	6A	XY-PAV.6A	3.73	3.68	1.22%
	6A	COM-PAV.6A	3.64	3.68	−1.18%
	5B	YN-PAV.5B	3.68	3.45	6.82%
	4J	4J	3.31	3.63	−8.71%
LWR	2A	YN-PAV.2A	1.91	2.01	−4.63%
	6A	YN-PAV.6A	2.00	1.84	8.48%
	6A	XY-PAV.6A	1.89	1.84	2.58%
	6A	COM-PAV.6A	1.86	1.84	0.89%
	1J^S^	1J^S^	2.00	1.93	3.62%
	4J	4J	2.14	1.91	11.88%
GD	2A	YN-PAV.2A	4.98	4.84	2.77%
	6A	YN-PAV.6A	4.85	4.97	−2.41%
	6A	XY-PAV.6A	5.04	4.97	1.38%
	6A	COM-PAV.6A	4.92	4.97	−1.13%
	5B	YN-PAV.5B	5.01	4.78	4.90%
	4J	4J	4.74	4.94	−3.96%
GC	6A	YN-PAV.6A	18.21	18.22	−0.08%
	6A	XY-PAV.6A	18.63	18.22	2.22%
	6A	COM-PAV.6A	18.13	18.22	−0.51%
	5B	YN-PAV.5B	18.62	17.91	3.95%
	1D	YN-PAV.1D	18.19	18.78	−3.11%
	1J^S^	1J^S^	18.08	18.29	−1.12%
	6J	6J	17.83	18.33	−2.75%
GA	6A	YN-PAV.6A	18.73	19.64	−4.61%
	6A	XY-PAV.6A	20.17	19.64	2.74%
	6A	COM-PAV.6A	19.23	19.64	−2.08%
	5B	YN-PAV.5B	19.95	18.15	9.87%
	2D	XY-PAV.2D	19.20	19.16	−0.19%
	4J	4J	17.91	19.38	−7.59%

Note: Data in the table represent the mean across three consecutive cultivation years: 2020–2021, 2021–2022, and 2022–2023. Effect value = (PAV − NC)/NC × 100%. NC: null control, the parental type line that lacks the specific fluorescent signals present in the other parental type.

**Table 3:** Oligo nucleotide probes for fluorescence in situ hybridization (FISH).

Probes	Modification Types	Probe Sequences
Oligo-B11	5′6-FAM	5′TCCGCTCACCTTGATGACAACATCAGGTGGAATTCCGTTCGAGGG3′
Oligo-pSc119.2	5′6-FAM	5′CCGTTTTGTGGACTATTACTCACCGCTTTGGGGTCCCATAGCTAT3′
Oligo-pTa535	5′TAMRA	5′AAAAACTTGACGCACGTCACGTACAAATTGGACAAACTCTTTCGGAGTATCAGGGTTTC3′
Oligo-(GAA)_10_	5′6-FAM	5′GAAGAAGAAGAAGAAGAAGAAGAAGAAGAA 3′
Oligo-pTa71	5′TAMRA	5′GGGCAAAACCACGTACGTGGCACACGCCGCGTA3′
Oligo-pAs1-1	5′TAMRA	5′GGATGCACTTCGTGTACAAAACGGACAATCTCTTTCAAAGTATCAGGATTTCATCC3′

## Data Availability

The datasets generated and/or analyzed during the current study are available from the corresponding author upon reasonable request.

## Supplementary Materials

The following supporting information can be downloaded at https://www.mdpi.com/article/10.3390/plants14193072/s1: Figures S1–S3: Karyotype of BC_1_F_4_–BC_1_F_6_-derived lines based on probe OMPM#4-M. Figure S4: Karyotype of special chromosome variations in BC_1_F_4_–BC_1_F_6_-derived lines. Letters a-k indicate the derivative lines 20WZ38, 20WZ23, 20WZ51, 20WZ52, 20WZ53, 20WZ29, 20WZ32, 20WZ41, 21WZ28, 22WZ31, and 22WZ32, respectively. Bar: 10 μm. Tables S1–S3: Chromosomal variations in BC_1_F_4_ -BC_1_F_6_-derived lines. Table S4: Association analysis between chromosomal variation and thousand-grain weight (TGW). Table S5: Association analysis between chromosomal variation and grain length (GL). Table S6: Association analysis between chromosomal variation and grain width (GT). Table S7: Association analysis between chromosomal variation and grain length/width ratio (LWR). Table S8: Association analysis between chromosomal variation and grain diameter (GD). Table S9: Association analysis between chromosomal variation and grain circumference (GC). Table S10: Association analysis between chromosomal variation and grain roundness (GR). Table S11: Association analysis between chromosomal variation and grain area (GA). Table S12. Grain traits of lines with special chromosomal variations in BC_1_F_4_–BC_1_F_6_-derived lines.
