# Supplementary material for: Impact of Alien Chromosome Introgression from Thinopyrum ponticum on Wheat Grain Traits
Source: Plants (Basel). 2025 Oct 4;14(19):3072. doi: 10.3390/plants14193072 (PMC12525785; doi:10.3390/plants14193072)
Supplement: Supplementary file 1 [file plants-14-03072-s001.zip › Supplementary Fig. S1-S4.pdf]

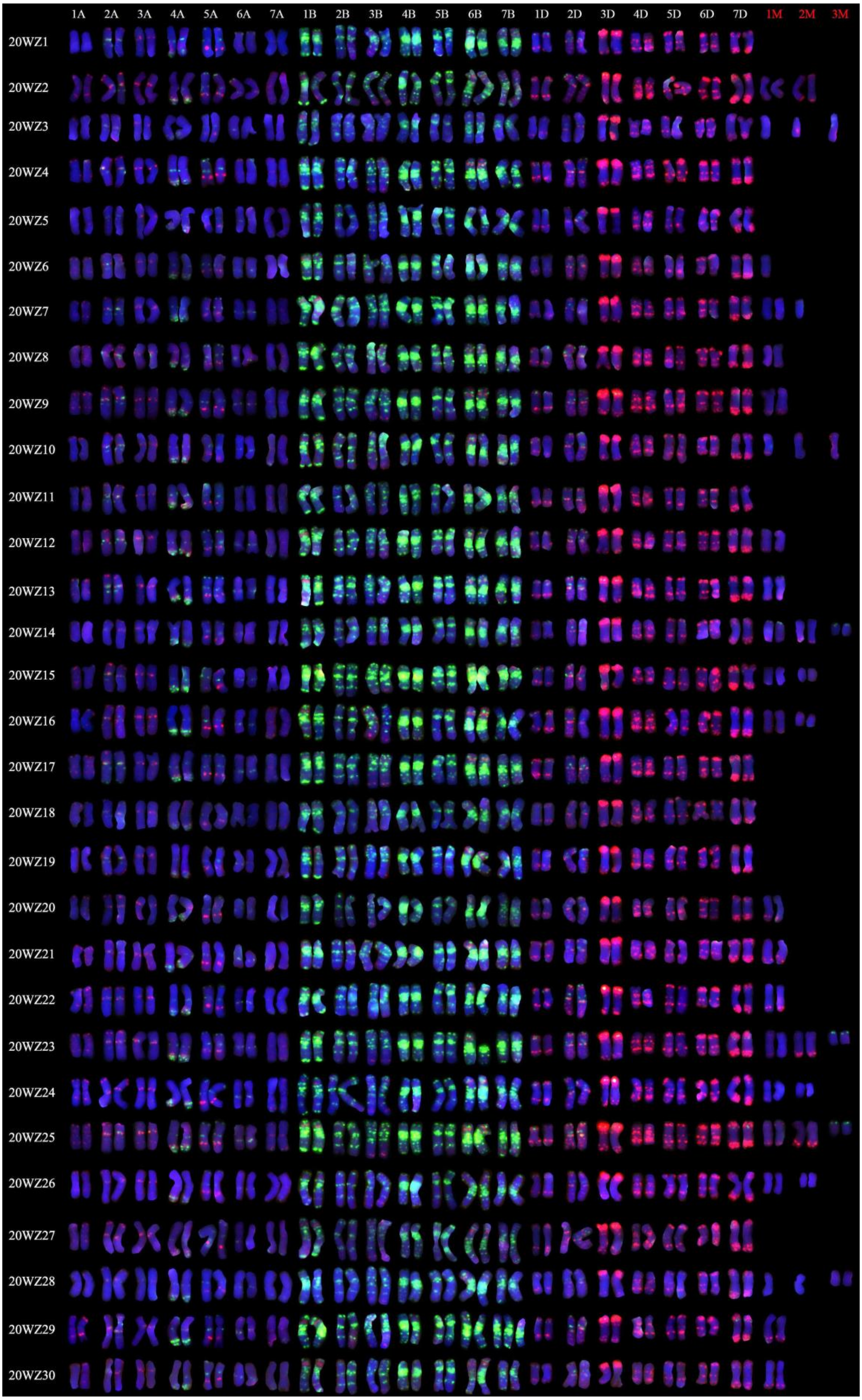
1A 2A 3A 4A 5A 6A 7A 1B 2B 3B 4B 5B 6B 7B 1D 2D 3D 4D 5D 6D 7D 1M 2M 3M
20WZ1
20WZ2
20WZ3
20WZ4
20WZ5
20WZ6
20WZ7
20WZ8
20WZ9
20WZ10
20WZ11
20WZ12
20WZ13
20WZ14
20WZ15
20WZ16
20WZ17
20WZ18
20WZ19
20WZ20
20WZ21
20WZ22
20WZ23
20WZ24
20WZ25
20WZ26
20WZ27
20WZ28
20WZ29
20WZ30

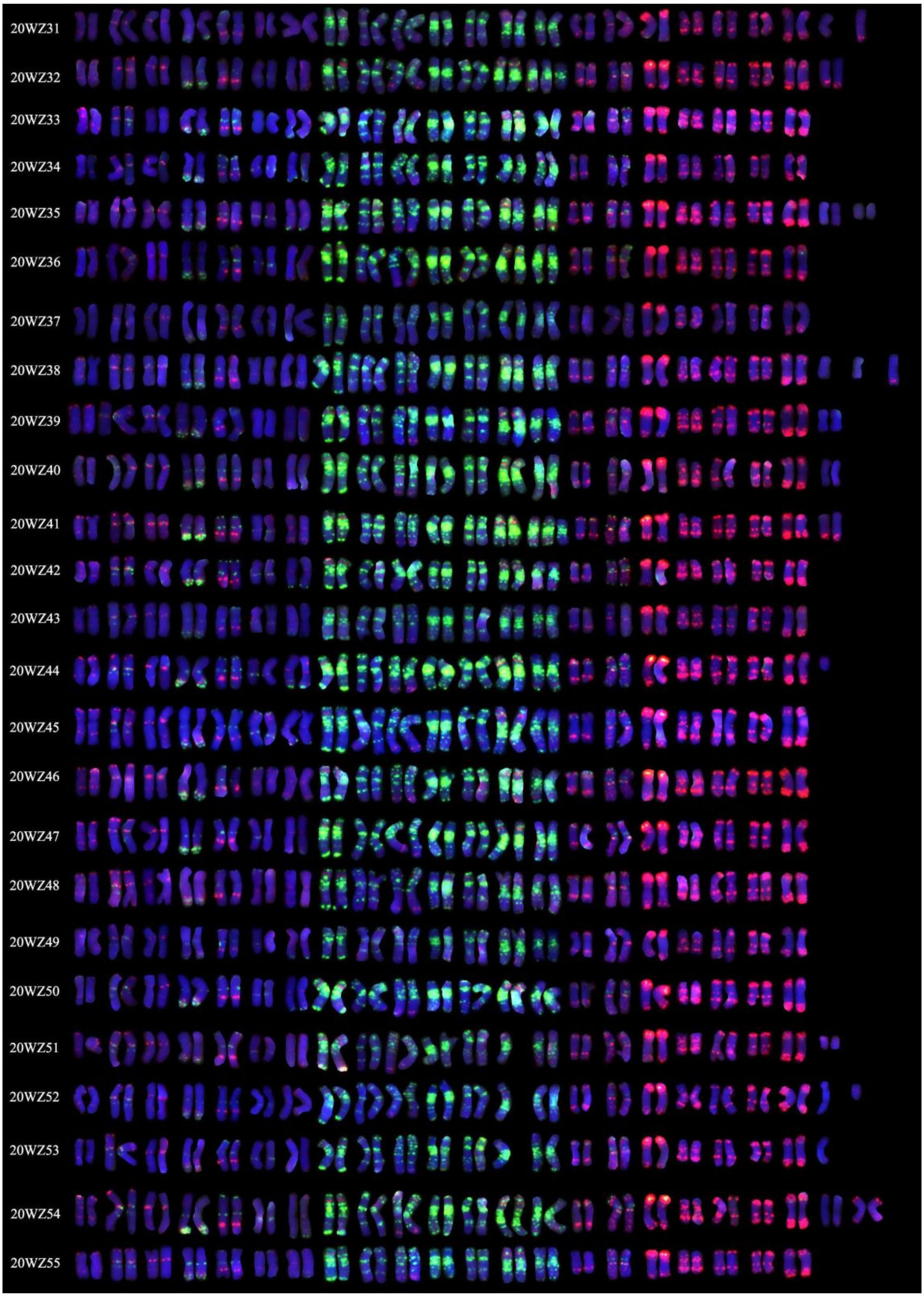

Fig. S1 Karyotype of BC_1F_4 derived lines based on probe OMPM#4-M.

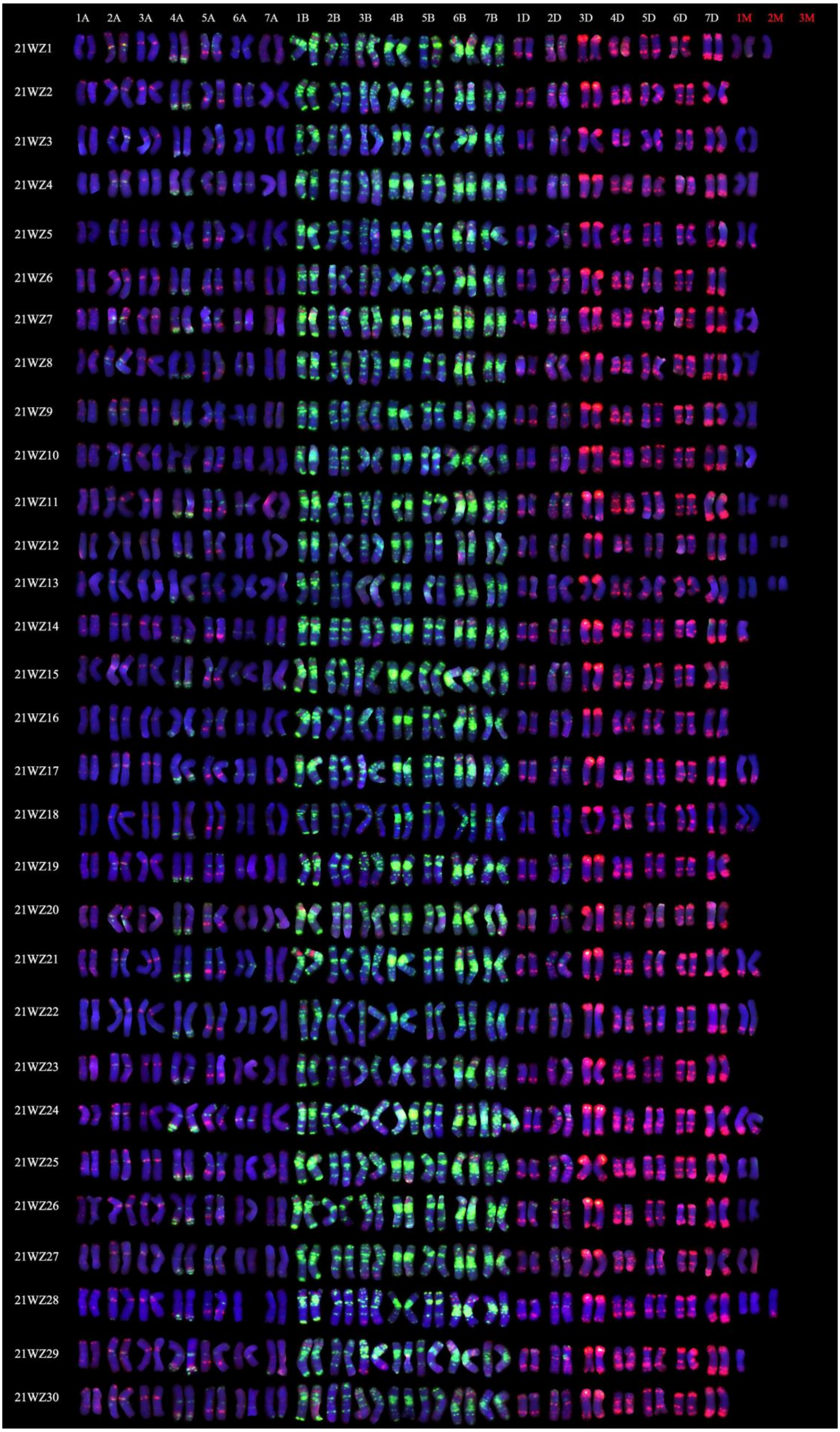
1A 2A 3A 4A 5A 6A 7A 1B 2B 3B 4B 5B 6B 7B 1D 2D 3D 4D 5D 6D 7D 1M 2M 3M
21WZ1
21WZ2
21WZ3
21WZ4
21WZ5
21WZ6
21WZ7
21WZ8
21WZ9
21WZ10
21WZ11
21WZ12
21WZ13
21WZ14
21WZ15
21WZ16
21WZ17
21WZ18
21WZ19
21WZ20
21WZ21
21WZ22
21WZ23
21WZ24
21WZ25
21WZ26
21WZ27
21WZ28
21WZ29
21WZ30

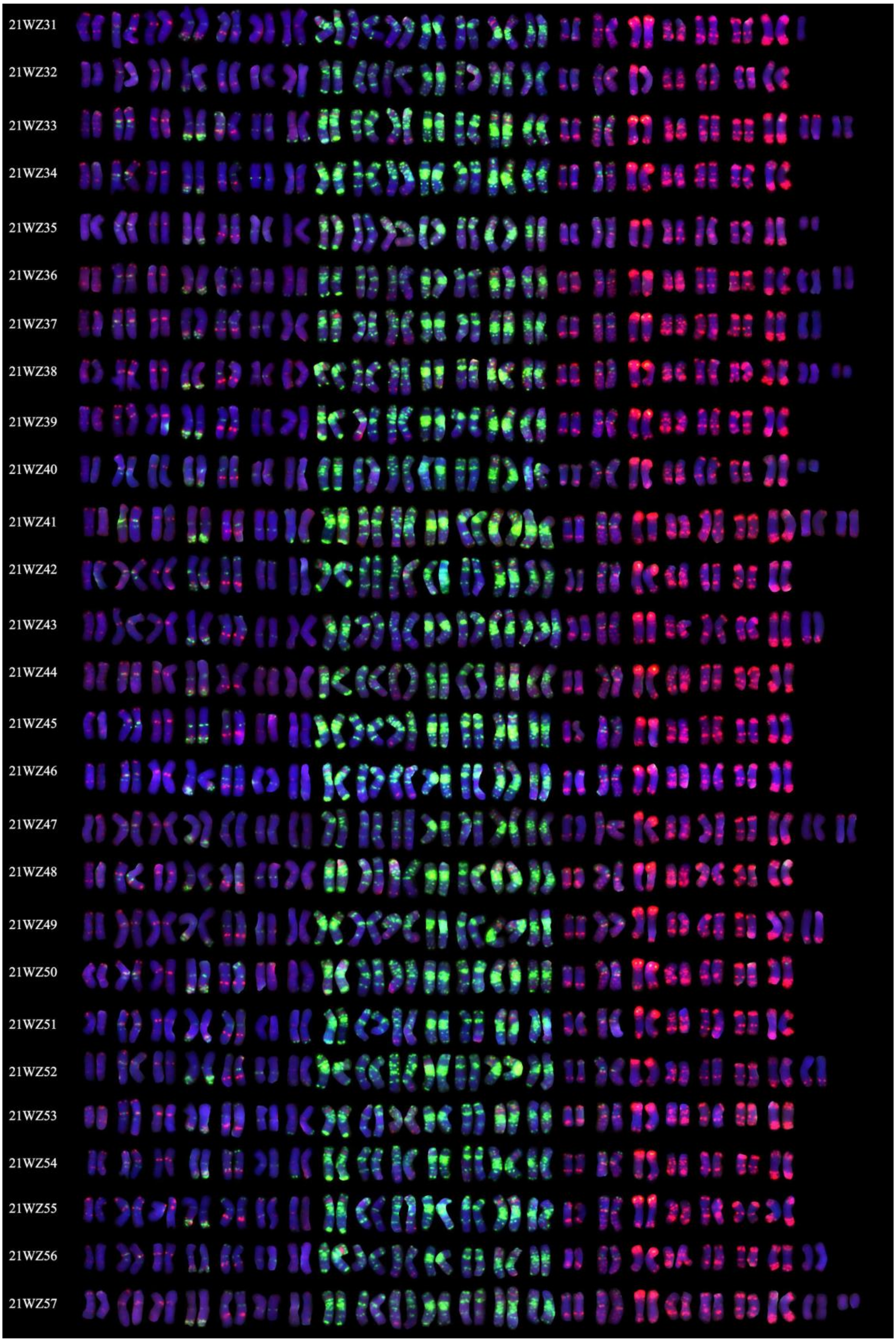

Fig. S2 Karyotype of BC_1F_5 derived lines based on probe OMPM#4-M.

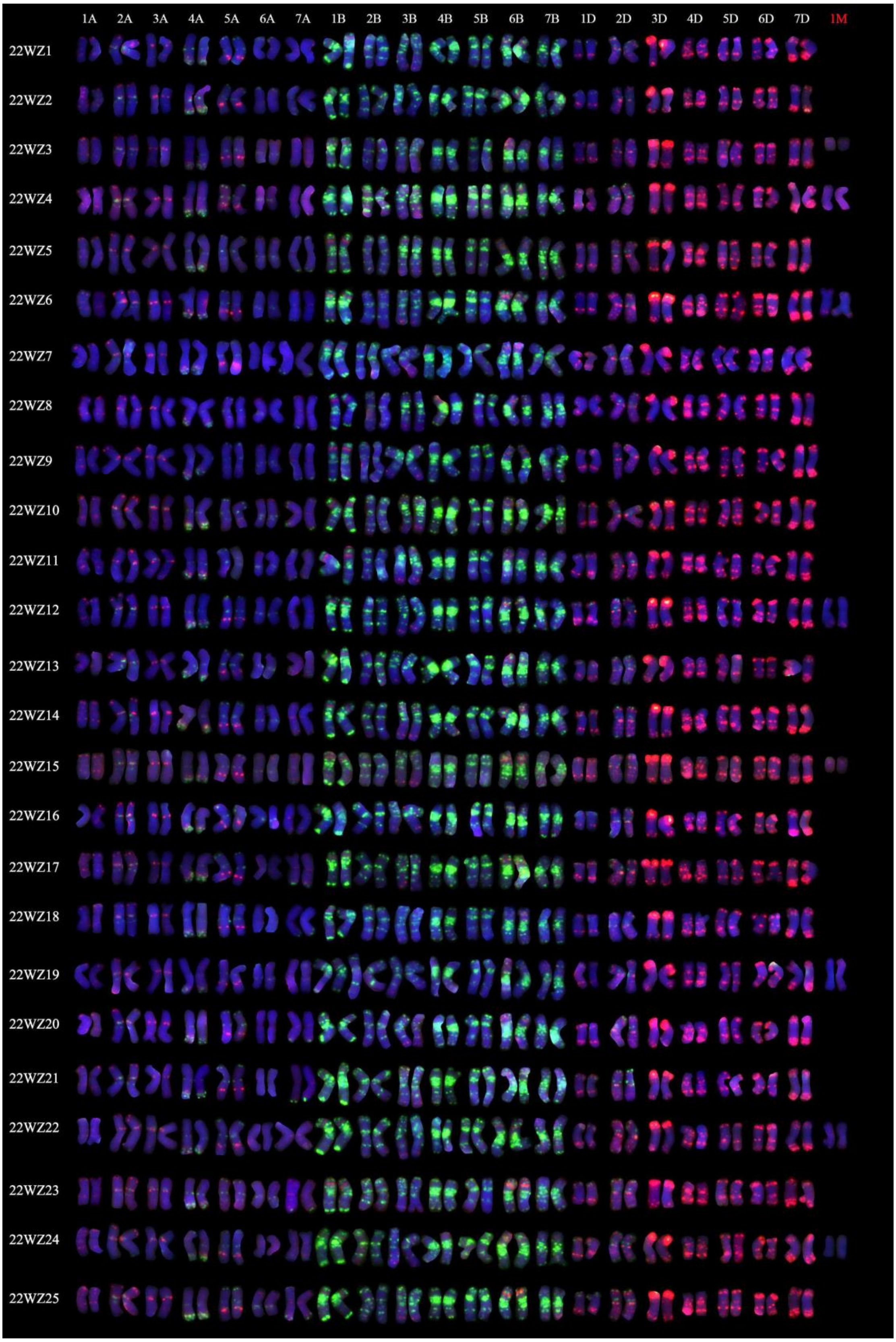

1A 2A 3A 4A 5A 6A 7A 1B 2B 3B 4B 5B 6B 7B 1D 2D 3D 4D 5D 6D 7D 1M
22WZ1
22WZ2
22WZ3
22WZ4
22WZ5
22WZ6
22WZ7
22WZ8
22WZ9
22WZ10
22WZ11
22WZ12
22WZ13
22WZ14
22WZ15
22WZ16
22WZ17
22WZ18
22WZ19
22WZ20
22WZ21
22WZ22
22WZ23
22WZ24
22WZ25

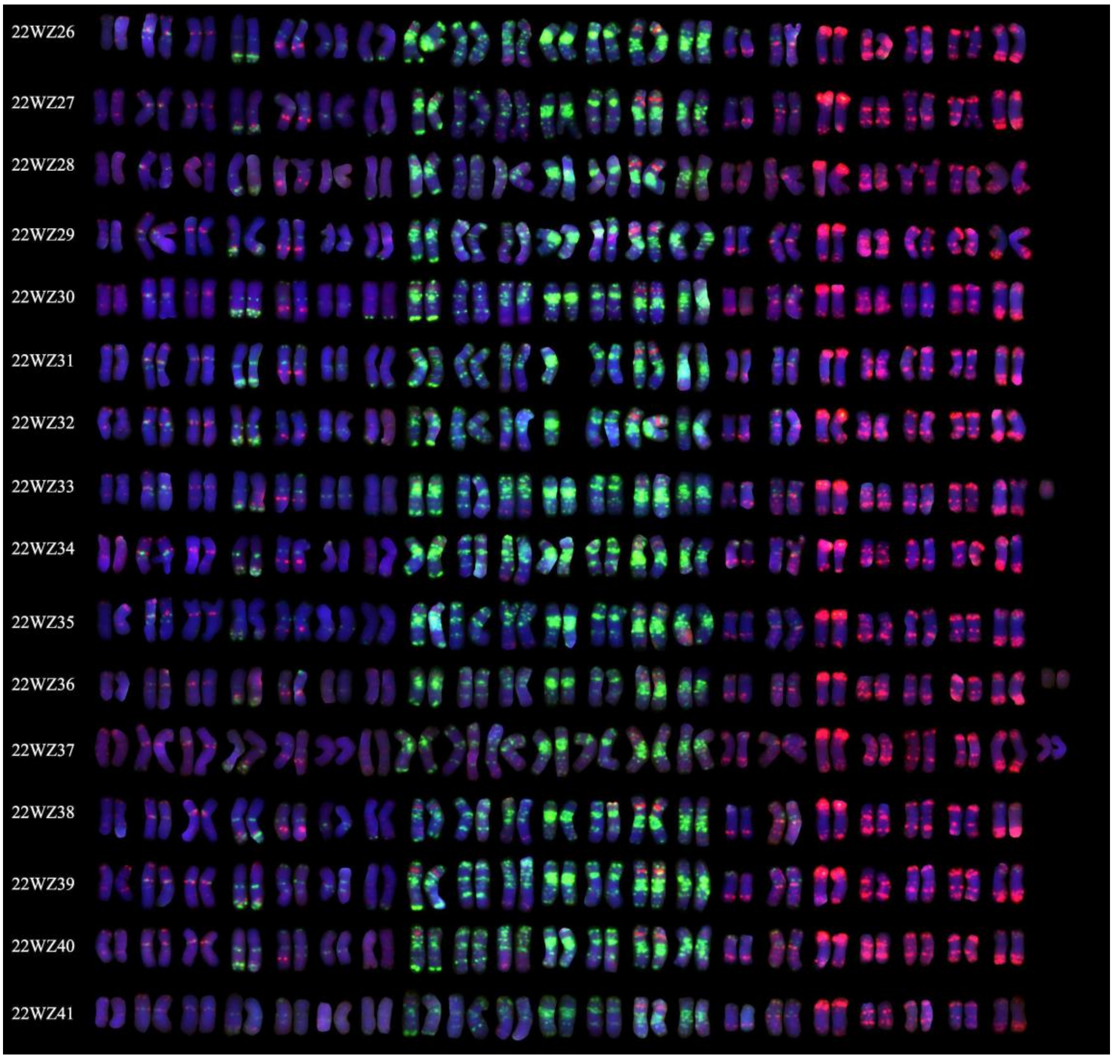

Fig. S3 Karyotype of BC_1F_6 derived lines based on probe OMPM#4-M.

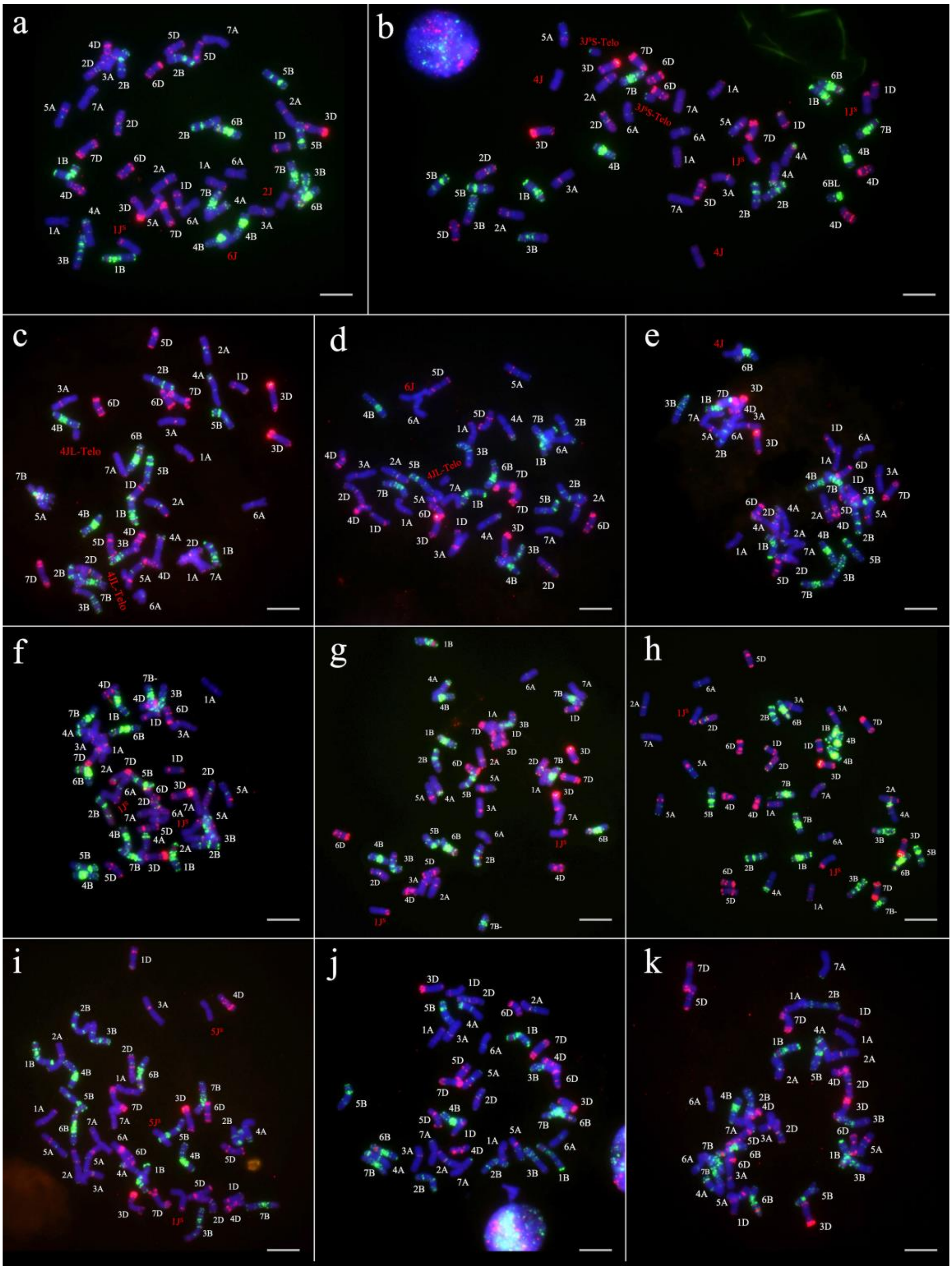

Fig. S4 Karyotype of special chromosome variations based on probe OMPM#4-M in the BC_1F_4 -BC_1F_6 derived lines. Letters a-k indicates the derivative lines 20WZ38, 20WZ23, 20WZ51, 20WZ52, 20WZ53, 20WZ29, 20WZ32, 20WZ41, 21WZ28, 22WZ31, and 22WZ32, respectively. Bar: 10 μm.
